# Effect of Protoberberine-Rich Fraction of *Chelidonium majus* L. on Endometriosis Regression

**DOI:** 10.3390/pharmaceutics13070931

**Published:** 2021-06-23

**Authors:** Alicja Warowicka, Badr Qasem, Anna Dera-Szymanowska, Maria Wołuń-Cholewa, Patryk Florczak, Nikodem Horst, Marta Napierała, Krzysztof Szymanowski, Łukasz Popenda, Grażyna Bartkowiak, Ewa Florek, Anna Goździcka-Józefiak, Piotr Młynarz

**Affiliations:** 1 Department of Animal Physiology and Development, Faculty of Biology, Adam Mickiewicz University in Poznań, 61614 Poznań, Poland; 2 NanoBioMedical Centre, Adam Mickiewicz University in Poznań, 61614 Poznań, Poland; patryk.florczak@amu.edu.pl (P.F.); lpopenda@amu.edu.pl (Ł.P.); gbartkow@amu.edu.pl (G.B.); 3 Department of Biochemistry, Molecular Biology and Biotechnology, Faculty of Chemistry, Wrocław University of Science and Technology, 50370 Wrocław, Poland; badr.qasem@pwr.edu.pl (B.Q.); piotr.mlynarz@pwr.edu.pl (P.M.); 4 Department of Perinatology and Gynecology, Poznań University of Medical Sciences, 60535 Poznań, Poland; annaszerszen@wp.pl; 5 Chair and Department of Cell Biology, Poznań University of Medical Sciences, 60806 Poznań, Poland; doskon@ump.edu.pl; 6 Department of General and Colorectal Surgery, Poznań University of Medical Sciences, 61285 Poznań, Poland; nvhorst@gmail.com; 7 Laboratory of Environmental Research, Department of Toxicology, Poznań University of Medical Sciences,60631 Poznań, Poland; martan@ump.edu.pl (M.N.); eflorek@ump.edu.pl (E.F.); 8 Department of Medical Education, Poznań University of Medical Sciences, 60806 Poznań, Poland; kp.szymanowski@wp.pl; 9 Molecular Virology Research Unit, Adam Mickiewicz University in Poznań, 61614 Poznań, Poland; agjozef@amu.edu.pl

**Keywords:** protoberberine compounds, berberine, *Chelidonium majus* L., endometriosis, metabolomics

## Abstract

Endometriosis is a gynecological disease defined by the presence of endometrial tissue outside the uterus. To date, the effective treatment of this disease is still based on invasive surgery or laparoscopy. *Chelidonium majus* L. (Papaveraceae) belongs to medicinal, latex-bearing plants. Extracts from the plant are a rich source of pharmacologically active agents. Protoberberine compounds derived from *C. majus* possess anticancer and antiproliferative activities. In the present study of a rat model of endometriosis, we investigated the influence of the plant protoberberine-rich fraction (BBR) obtained from the medicinal plant *C. majus* on the development of endometriosis. To understand of BBR therapeutic potential for endometriosis, metabolomics has been applied to study. BBR was prepared from an ethanolic extract of dry plants *C. majus*. Rats (*n* = 16) with confirmed endometriosis were treated with BBR administered orally (1 g/kg) for 14 days. Blood serum samples were collected from all of the animals and metabolites were studied using the NMR method. The metabolomic pattern was compared before and after the protoberberine treatment. The performed analysis showed significant changes in the concentrations of metabolites that are involved in energy homeostasis, including glucose, glutamine, and lactate. Histopathological studies showed no recurrence of endometriosis loci after treatment with BBR. The results of the study found that BBR treatment prevents the recurrence of endometriosis in rats. Moreover, metabolomics profiling can be applied to better understand the mechanisms of action of these protoberberine secondary plant metabolites. Our findings provide new insights into the pharmaceutical activity of natural protoberberine plant compounds.

## 1. Introduction

Endometriosis is a common disorder affecting women of reproductive age. The disease affects approximately 10% of reproductive women. The disease can cause chronic pelvic pain, dysmenorrhea, dyspareunia, and reduced fertility. Endometriosis is characterized by the growth of endometrial glands and stroma outside of the uterus [1]. The etiology of the disease is unknown, and no single theory can explain its pathogenesis. It has been shown that key cellular processes such as apoptosis, proliferation, attachment, and cytoskeleton organization are disrupted in endometriotic cells [2]. Many studies suggest that it is a polygenic–multifactorial type of pathogenic state, the phenotype of which is determined by a combination of multiple genes, sex steroid hormone signaling, and epigenetic and environmental factors. Recently, meta-analyses identified five novel loci associated with endometriosis. These genes are involved in hormone metabolism [3]. Numerous studies have indicated that immunological factors also play an important role in the pathogenesis of endometriosis and endometriosis-associated infertility. A recent study described that the cell-mediated immune response contributes to the growth of endometrial tissue in implantation sites. In endometriosis-suffering women, aberrant changes are shown in the number and activation of peritoneal macrophages. Additionally, T-cell reactivity, natural killer cell cytotoxicity, increased circulating antibodies, the presence of autoantibodies, and changes in the cytokine network have been observed [4]. The diagnosis of endometriosis is based on pelvic examination, diagnostic ultrasound, laparoscopy, and magnetic resonance imaging (MRI). A common serum cancer antigen 125 (CA125), is also detected and used for non-invasive diagnosis. Unfortunately, the marker is efficient in only the diagnosis of the advanced stages of endometriosis [5]. Recently, metabolomics profiling has been recognized as a powerful tool for elucidating the pathogenesis of endometriosis and for disease biomarker identification. Nuclear magnetic resonance (NMR)-based and mass spectrometry (MS)-based metabolomics studies on endometrial fluid, follicular fluid, urine, and the serum and plasma of endometriosis-suffering women have shown changes in their metabolite profiles [6,7].

Endometriosis is a gynecological disorder that is difficult to treat. Currently, there is no good cure for this disease, and its treatment usually involves surgery. Applied medicines that inhibit estrogen receptors are mainly based on treating endometriosis-typical symptoms rather than healing the disease. Moreover, medical hormonal treatment causes side effects. To date, many studies have shown that Chinese medicine (CM) therapy, which usually uses a mixture of medicinal herbs, can produce good results in endometriosis treatment. Each of the plants is likely composed of several active components, which act synergistically and have anti-inflammatory, anti-proliferative, and pain-alleviating properties. For decades, a variety of medicinal herbs have been used for endometriosis treatment—e.g., cnidium fruit, corydalis, curcuma, frankincense, myrrh, or white peony root. These medicinal herbs are the source of many natural compounds, such as triterpenoids, flavone polyphenolic compounds, essential oils, coumarins, triterpene acids, alkaloids, and others [8].

One of the bioactive plant components that has become popular is berberine. It is a benzylisoquinoline alkaloid with many pharmacological activities involving multiple biological mechanisms. Berberine has strong anti-inflammatory and anti-cancer properties. It has been reported that this alkaloid shows anti-proliferative effects on cancer cells, with minimal effects on normal cells [9]. Furthermore, some reports show that berberine has anti-angiogenic activities and inhibits angiogenesis by downregulation of vascular endothelial growth factor receptor (VEGFR) mRNA expression [10]. Furthermore, berberine is a natural DNA intercalator that forms strong complexes with DNA and RNA. Noureini et al. demonstrated that berberine obtained from medicinal herb *Chelidonium majus* L. strong interacts with G-quadruplex and inhibits telomerase activity [11]. Because of these significant pharmacological properties, berberine and its derivatives have been utilized for the treatment of various diseases, including diabetes and various types of cancer [12]. In recent years, berberine has become popular for relieving the symptoms of several gynecological disorders, including endometriosis [13]. Nevertheless, a detailed molecular mechanism of action has not yet been described.

In the present study of a rat model of endometriosis, we examined the influence of the plant protoberberine-rich fraction (BBR) obtained from the medicinal plant *C. majus* on the development of endometriosis. For this purpose, we investigated the metabolomic profile changes in endometriosis upon treatment of the disease with the studied BBR.

## 2. Materials and Methods

### 2.1. Plant Material and Preparation of Natural Alkaloidal Protoberberine-Rich Fraction from C. majus

For the present study, dry *C. majus* plants were used. The natural alkaloidal fraction was extracted from *C. majus* as described previously, with modifications [14]. A stock solution of the sample was prepared by dissolving it in dimethyl sulfoxide (DMSO). First, 200 g of dried plant material (*C. majus*, Flos) was soaked in 2 L of 96% ethanol (POCH). The mixture was boiled in a reflux condenser for 12 h. After cooling down to room temperature (12–24 h), the mixture was filtered using filter paper. The obtained ethanolic extract was evaporated under reduced pressure at a temperature lower than 40 °C in a rotary evaporator. The residues were dissolved in hot distilled water (100 mL), sonicated (around 10 min), and filtered (sintered glass filtration, Labit G-3). Next, in order to precipitate the berberine alkaloids in the form of their chlorides, the appropriate amount of 36% HCl was added to the filtrate in a volume ratio of 1:3. A dark brown precipitate was formed after 7–10 days in a refrigerator (5 °C). To separate the protoberberine extract, the precipitate was centrifuged (24,000 rpm, 10 °C, 50 min) and dried in a vacuum at 60 °C for 3 h, using a vacuum concentrator (1600 rpm).

### 2.2. Identification of Compounds by HPLC/ESI-MS and NMR

For the exact determination of the composition of the fraction from *C. majus*, the samples used for in vivo studies were analyzed using high-performance liquid chromatography–mass spectrometry (HPLC/ESI-MS) and nuclear magnetic resonance (NMR) techniques. The HPLC/ESI-MS/MS analyses were performed using an UltiMate 3000 UHPLC System (Thermo Fisher Scientific, Waltham, MA, USA) equipped with a Kinetex C18 column (Phenomenex, 2.1 × 100 mm, 2.6 μM) and impact HD (ESI-QTOF) mass spectrometer (Bruker Daltonics, Bremen, Germany). The HPLC/ESI-MS/MS analyses were performed using an UltiMate 3000 UHPLC System (Thermo Scientific) equipped with a Kinetex C18 column (Phenomenex, 2.1 × 100 mm, 2.6 μM) and impact HD (ESI-QTOF) mass spectrometer (Bruker Daltonics, Bremen, Germany). The purity of the studied alkaloidal fraction, determined on the basis of the HPLC chromatogram (Supplementary Figure S1a) was >98%. The HPLC/ESI-MS analyses were carried out as described previously [15]. The NMR spectra were recorded at 298 K by means of an Agilent DD2 800 MHz spectrometer equipped with a 5 mm 1H/13C/15N triple-resonance probe head. The resonance assignments were made on the basis of ^1^H, ^1^H-^1^H COSY, ^1^H-^1^H TOCSY, ^1^H-^13^C HSQC, and ^1^H-^13^C HMBC experiments. The identification of berberine, coptisine, chelidonine, and sanguinarine in the extract was confirmed by comparison of obtained NMR spectral data with those collected for reference standards. Stylopine was identified referring to data reported by Kim et al. [16]. The presence of all remaining compounds was confirmed basing on data collected in the Human Metabolome Database [17] and the Aldrich library of ^13^C and ^1^H FT-NMR spectra [18].

### 2.3. In Vivo Study Design: Autotransplantation of Uterine Squares to the Peritoneal Cavity and Pharmacological Treatment

Thirty-nine mature female Wistar white rats (weighing 180–220 g) were purchased from the Animal Center Laboratory of the University of Medical Sciences (Poznan, Poland). The animals were maintained in the Department of Toxicology, University of Medical Sciences in Poznan in an environmentally controlled area, and maintained with water and food ad libitum. The room temperature and relative air humidity were set at 24 ± 1 °C and 50 ± 5%, respectively, with a 12-hour light–dark cycle. Before the experiment, all of the rats were acclimated to the laboratory conditions for seven days. The rats were randomly divided into three groups, namely the control group (sham group, HC-SHO; *n* = 9), the model group (surgery group, E-C; *n* = 9), and the protoberberine-rich fraction (BBR) treatment group (surgery group, E-BBR; *n* = 21).

The experiments were approved on 4 September 2017, the Institutional Review Board (IRB) of Poznan University of Life Sciences. The protocols used in the study were approved by the Regional Animal Research Committee no. 42/2017, and all procedures were carried out in accordance with the Helsinki Declaration.

In this study, the model of recurrent rat endometriosis was based on autologous endometrium graft procedures previously described by Szymanowski et al. in 2013 [19]. Endometriosis induction was performed in the model group (E-C) and the treatment group (E-BBR) on rats exhibiting four or more consecutive estrous cycles of four or five days. Under pentobarbital anesthesia (sodium pentobarbital, 3%), a first mid-ventral laparotomy surgery was performed aseptically. Using microsurgical techniques, 3 cm segments of the right and left uterine horns were ligated and excised. The removed horns were placed in sterile culture medium and cut into two parts for further experiments. The endometrium was detached from the muscular layer, and formed into two grafts each measuring 3 mm × 3 mm. These two pieces were then attached to the parietal peritoneum on the left and right sides of the abdominal wall. Sutures of 6-0 nylon were used to attach the grafts. The abdominal wall was closed with a 1-0 Vicryl running suture. In the control group with sham operation (HC-SHO), only the abdominal incision was carried out—uterine tissue autotransplantation was not performed. Following the surgery, the treatment group (E-BBR) was orally administrated protoberberine-rich fraction (BBR) at a dose of 1 g/kg for 14 days via oral gavage, while a vehicle (0.9% NaCl) was supplemented to the model group (E-C) and the control group (HC-SHO).

### 2.4. Evaluation of Ectopic Endometrium Loci Formations: H & E Staining

After the autotransplantation of the uterine squares to the peritoneal cavity of rats, a second laparotomy surgery was performed to assess the ectopic endometrium loci formations and adhesions. The animals were anesthetized with sodium pentobarbital (3%), as mentioned above (Section 2.3). The suspected lesions (the presence of cysts filled with fluid and/or the presence of adhesion around the implant, and/or clearly visible hypervascularisation on the abdominal peritoneum) and the eutopic endometrium were excised for histopathological analysis. For further research, we included only the rats that showed the development of endometriosis into highly vascular, tear-shaped cysts with blood and serous fluid. A third laparotomy was performed after the euthanasia of all of the animals. Once again, after three months, we searched for endometriosis foci, and the suspected lesions in the surgery groups (E-C and treatment E-BBR groups), as well as the eutopic endometrium, were excised for histology.

All of the tissue fragments acquired during the laparotomies were fixed in 4% formaldehyde, embedded in paraffin, sectioned on a microtome, and stained with hematoxylin and eosin (H & E staining) for routine examination.

### 2.5. Collection and Preparation of Serum Samples for NMR Analysis

Blood samples (5 mL of heart blood) were collected from all of the rats for the metabolome profile characterization. The blood samples were subsequently centrifuged (2000× *g*, 10 min, 4 °C), and the serum samples were transported in dry ice to Wrocław University of Science and Technology (Prof. Mlynarz’s laboratory) and then stored in −80 °C until further use. Before the NMR analysis, the samples were thawed at room temperature; vortexed; and 200 μL was taken and mixed with 400 μL of NaCl 0.9% saline solution, 15% D_2_O, and 3 mM TSP. Next, the samples were centrifuged at 12,000 rpm for 10 min. After centrifugation, we transferred 550 μL from the supernatant to a 5 mm NMR tube.

### 2.6. ^1^H NMR Spectroscopic Measurements

The serum samples were recorded on a Bruker AVIII 600 spectrometer (Bruker, Karlsruhe, Germany) at 300 K. The operation was at a proton frequency of 700.48 MHz. The ^1^H NMR spectra were recorded by using a CPMG pulse sequence with water presaturation (*cpmgpr1d* in Bruker notation) to detect low-molecular-weight metabolites over a spectral width of 14.99 ppm with 128 transients, the time domain was 65 k, with an acquisition time of 3.12 s, and 3.5 s relaxation delay.

The process of spectra were first with a line broadening of 0.3 Hz and manually were phased and baseline corrected using Topspin 3.2 software (Bruker BioSpin, Rheinstetten, Germany). The reference of spectra were an α-glucose signal (δ  =  5.222 ppm). The signal alignment process done by using the correlation optimized warping algorithm (COW) [20] and by using MATLAB (v 8.3, Mathworks Inc. Natick, MA, USA) to implement *icoshift* algorithm [21]. The water spectrum region was removed from the calculations. All of the spectra were normalized using the Probabilistic Quotient Normalization (PQN) method [22,23].

### 2.7. ^1^H NMR Identification of Metabolites for Serum Samples

The identification of metabolite resonances was obtained according to the assignments published in the literature and databases on-line (Biological Magnetic Resonance Data Bank and Human Metabolome Data Base) (Table S3).

### 2.8. Multivariate Data Analysis

The multivariate analysis was conducted for spectral integral values of metabolites by using SIMCA software (version 17.0, Umetrics, Sartorius, Göttingen, Germany). Orthogonal partial least squares-discriminant analysis score plots (OPLS-DA) was used to show the differences between the groups of the ^1^H NMR data, after choosing variables important for the VIP plot with a jack-knifed confidence interval. A confidence higher than 1.0 was obtained and, for VIP, we removed anything below a score of 1.0 from analysis and built a new OPLS-DA model; the reliability of the model was tested by A coefficient of variation-analysis of variance (CV-ANOVA) approach, and a *p*-value less than 0.05 was considered significant (Figure 4).

### 2.9. Statistical Analysis

For each metabolite of the rat serum samples, relative standard deviations (RSD) for independent groups were used to obtain the *p*-value (*p* < 0.05), calculated using STATISTICA13. Statistical significance based on the Mann–Whitney–Wilcoxon (*p* < 0.05) or Student’s *t*-test (*p* < 0.05) was calculated. The percentage difference (PD%) was calculated based on the mean values of relative signal integrals in each group.

For in vivo BBR treatment experiment, significant treatment effects used to compare the results between the control and study group were analyzed using a chi-square test. Calculated *p*-values less than 0.05 indicated statistically significant differences.

## 3. Results

### 3.1. Identification of Compounds in BBR by HPLC-ESI/MS and NMR

The natural BBR fraction was extracted from *C. majus*. HPLC/ESI-MS analysis showed the presence of compounds belonging to the protoberberine class of alkaloids, such as berberine, coptisine, dihydroberberine, stylopine, and protopine. Additionally, chelidonine and a small amount of allocryptopine and magnoflorine were detected in the obtained fraction. NMR confirmed the presence of protoberberine alkaloids—coptisine, berberine, sanguinarine, and stylopine. A detailed analysis of the MS, HPLC, and NMR spectra is presented in Supplementary Materials (Figures S1–S7; Tables S1 and S2).

### 3.2. In Vivo Effect of Surgical and Pharmacological Treatment: Evaluation of Ectopic Endometrium

**Figure 1:**
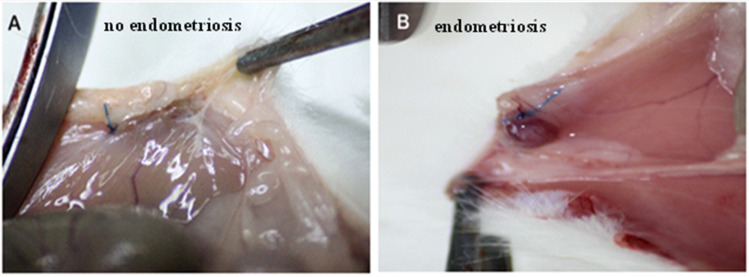
No signs of endometriosis on abdominal peritoneum—non-absorbable nylon sutures may be observed (**A**). Endometriosis on the abdominal peritoneum—highly vascular, tear-shaped cyst with blood and serous fluid (**B**).

The surgical induction of peritoneal endometriosis via autotransplantation was performed in 30 rats. All of the suspected endometriotic foci were confirmed by histological assessment. After the second laparotomy, it was found that five animals in the E-BBR group had not developed endometriosis (83%; Figure 1A). For further research using the E-BBR group, we included only those rats (*n* = 16) that showed the development of endometriosis into highly vascular, tear-shaped cysts with blood and serous fluid (Figure 1B). After the third relaparotomy, the development of endometriosis was revealed in 12.5% of rats from the protoberberine treatment group (E-BBR) and 61% in the model E-C group (*p* = 0.02). There were no other localizations of endometriosis than parietal peritoneum at implantation sites. The stage was assessed as early endometriosis in 80% I-II degree; ASRM classification) and in 20%-as III degree.

### 3.3. Histopathological Observations/Evaluation

**Figure 2:**
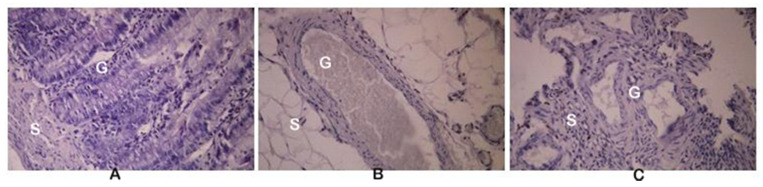
Histological sections of the eutopic endometrium (**A**), endometriosis (**B**) isolated from rats formed protoberberine treatment group (E-BBR), and endometriosis isolated from the model E-C group (**C**). G: gland; S: stroma; magnification 200×.

For the histopathological observations, all of the resected endometriosis tissues were fixed, embedded in paraffin, sectioned, and stained with hematoxylin and eosin (H & E staining). Histologically, the endometriosis implants (Figure 2C) were closely similar to the eutopic endometrium (Figure 2A), but only in the model E-C group. In the E-BBR group that received the BBR characteristic for the recurrence of endometriosis, atrophy of the stromal cells and the presence of endometrial glands were found (Figure 2B).

### 3.4. Metabolomic Analysis: Identification of Metabolites

**Figure 3:**
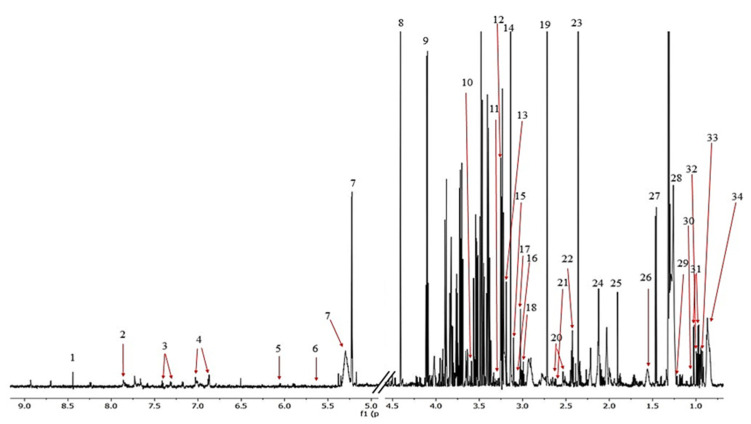
The representative ^1^H NMR spectrum obtained from the serum samples of the rats. The following metabolites were identified: (1) formate, (2) histidine, (3) phenylalanine, (4) tyrosine, (5) cytidine, (6) urea, (7) L_1, (8) glucose, (9) unknown_1, (10) lactate, (11) glycerol, (12) proline, (13) betaine, (14) choline, (15) malonate, (16) creatinine, (17) creatine, (18) lysine, (19) dimethylamine, (20) citrate, (21) methionine, (22) glutamine, (23) pyruvate, (24) acetone + lipid, (25) acetate, (26) L2, (27) alanine, (28) L3, (29) 3-hydroxybutyrate, (30) isobutyrate, (31) valine, (32) isoleucine, (33) leucine, and (34) L4.

**Figure 4:**
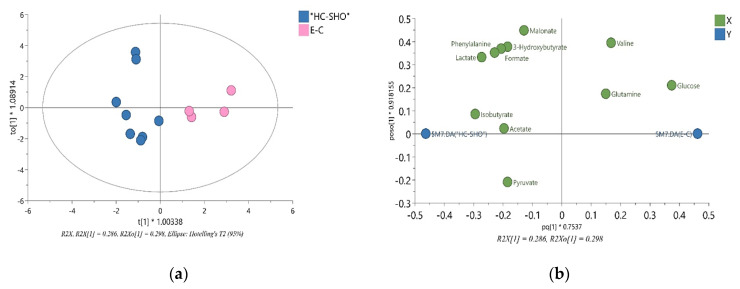
The VIP-OPLS-DA models plot for EC and HC-SHO groups. (**a**) The Score plot for the orthogonal partial least squares-discriminant analysis (OPLS-DA) model based on the eleven most important metabolites built with VIP value more than 1.0 (glucose, lactate, isobutyrate, phenylalanine, formate, 3-hydroxybutyrate, valine, malonate, pyruvate, acetate and glutamine) and all variables used in the analysis (R^2^X (cum); Goodness of fit (R^2^Y (cum)); goodness of prediction (Q^2^(cum)) and coefficient of variation (CV) Anova p-value showed in Table 1. (**b**) The VIP-OPLS-DA loading plot corresponding to the eleven most important metabolites with VIP value more than 1.0 obtained from ^1^H NMR data set. Pink color = EC group, Blue = HC-SHO group, VIP = variable importance in projection.

The ^1^H NMR spectra allowed 34 metabolites to be assigned and described (Figure 3). Initially, a PCA analysis was performed and did not shown significant grouping between all three of the investigated subjects. Next, the OPLS-DA analysis were performed, showing a model validation parameter *p*-value higher than 0.05 for each comparison except in comparison between EC and HC-SHO groups the model EC vs. HC-SHO did pass the validation by CV-ANOVA test (Figure 4, Table 1) after the VIP score comparison between groups demonstrates the differences by the importance of single metabolites in the model variance explanation and removing all metabolites with VIP value less than 1 and rebuild the model (Figure 4).

**Figure 5:**
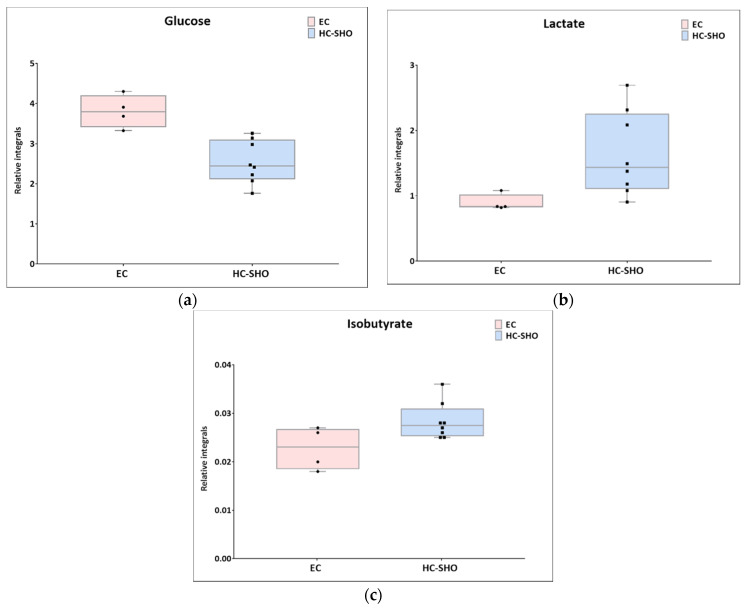
Boxplots for metabolites with statistically significant (*p* < 0.05) showed the metabolites changes in comparisons between EC and HC-SHO groups. (**a**) Glucose. (**b**) lactate. (**c**) isobutyrate. Pink bars–EC, blue bars–for HS-SHO, Whiskers–1.5 × interquartile range (IQR); bar–average; box–range between first quartile (Q1) and third quartile (Q3). Black circle–data point for EC, black square–data point for HC-SHO.

**Figure 6:**
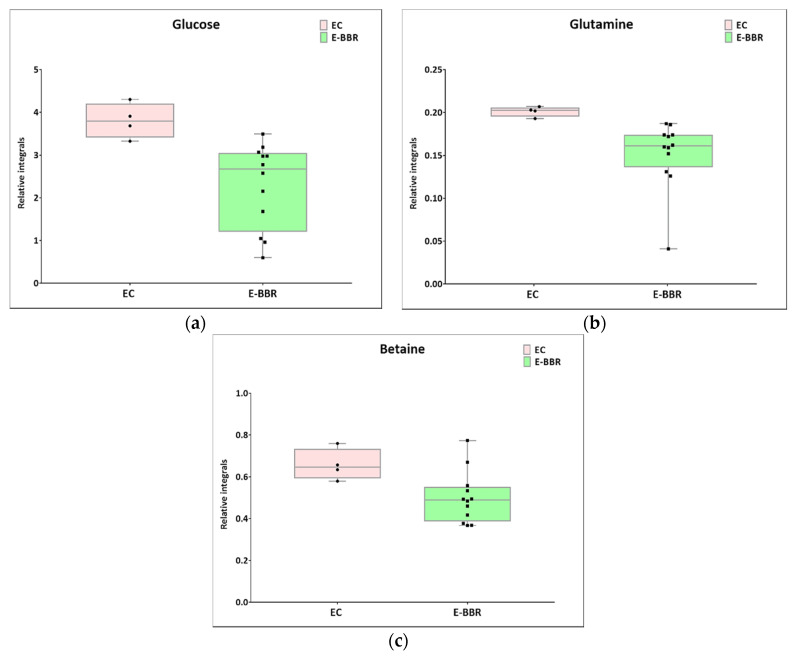
Boxplots for metabolites with statistically significant (*p* < 0.05) showed the metabolites changes in comparisons between EC and HC-SHO groups. (**a**) Glucose. (**b**) Glutamine. (**c**) Betaine. Pink bars–EC, green bars–for E-BBR, Whiskers–1.5 × interquartile range (IQR); bar–average; box–range between first quartile (Q1) and third quartile (Q3). Black circle–data point for EC, black square–data point for E-BBR.

**Figure 7:**
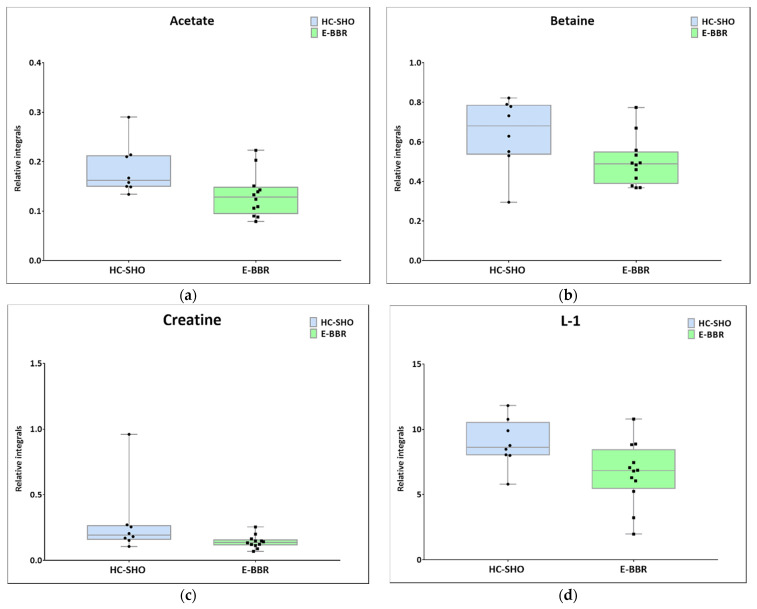
Boxplots for metabolites with statistically significant (*p* < 0.05) showed the metabolites changes in comparisons between EC and HC-SHO groups. (**a**) Acetate. (**b**) Betaine. (**c**) Creatine. (**d**) L-1_VLDL (0.84 ppm). Blue bars–HC-SHO, green bars–for E-BBR, Whiskers–1.5 × interquartile range (IQR); bar–average; box–range between first quartile (Q1) and third quartile (Q3). Black circle–data point for HC-SHO, black square–data point for E-BBR.

Next, univariate analysis was performed to find out the potential statistically important metabolites able to differentiate the studied groups (Table 2, Table 3 and Table 4). The HC-SHO vs. E-C comparison was expected to reveal the effect of endometriosis on serum metabolome, where only three metabolites were found to be perturbed. Glucose was downregulated in HC-SHO group along with upregulated lactate and isobutyrate (Table 2, Figure 5). The E-BBR vs. E-C comparison showed the direct influence of a protoberberine-rich fraction therapeutic effect, where glucose, glutamine, and betaine were of statistical significance (Table 3, Figure 6). The last analysis showed the differences between the healthy control and the protoberberine-treated rats (HC-SHO vs. E-BBR), showing the potential molecular proximity between these two groups and a systemic protoberberine influence on rats’ bodies. Among all of the metabolites found, four were statistically significant—acetate, creatine, betaine, and L1 (Table 4, Figure 7).

## 4. Discussion

Endometriosis is a common and complex disease characterized by the presence of endometrial gland and stroma tissue at ectopic sites. The etiology of endometriosis is unknown, and there are no specific markers for its diagnosis. There have been some studies describing an association between endometriosis and cancer. For example, a close relationship was observed between endometriosis and ovarian cancer [24]. Therefore, searching for novel diagnostic biomarkers of endometriosis is urgent. At present, the popular clinical serum marker—CA125 is efficient in only the diagnosis of the advanced stages of endometriosis (stages III–IV), and has a poor diagnostic value for indicating mild endometriosis (stages I–II), with a sensitivity of only of 24% [25]. Nowadays, different omics-based analyses are carried out in order to identify the biomarkers of endometriosis. One of these analyses is metabolomics, which provides a powerful approach to explore novel diagnostic biomarkers by analyzing changes in metabolomic profiles [6]. Previous metabolomics studies have shown that concentrations of energy, ketogenic, and glycogenic metabolites are significantly changed in the different stages of endometriosis. A previous study demonstrated that serum amino acids—such as proline, alanine, lysine, phenylalanine, and leucine—could be suitable molecules for endometriosis diagnosis [25]. Moreover, in women with endometriosis, an increased serum level of the 11 metabolites involved in the pyruvate metabolism and oxidative stress was also observed [26]. Besides the diagnostics of endometriosis, the treatment of this disease is also mainly based on laparoscopy and hormone therapy. To date, no satisfactory therapeutic options have reported in the management of endometriosis. Thus, it is urgent to search for a novel or adjuvant strategy for endometriosis treatment.

Berberine is a natural isoquinoline plant alkaloid extracted from medicinal herbs such as *Coptis chinesis*, *Amurense phellodendron*, and *Berberis vulgaris*. In recent years, the alkaloid has been used to reduce blood glucose and regulate blood lipids [12]. In our previous studies, we observed strong anti-proliferative activities of berberine and its derivatives extracted from *C. majus*, one of the most commonly used herbs in folk medicine for the treatment of viral infections, bacterial infections, and cancer [15]. The plant is a rich source of protoberberine alkaloids such as berberine, dihydroberberine, coptisine, sanguinarine, and protopine. Several recent reports have indicated that coptisine and sanguinarine have various biological activities and show anti-proliferative properties [27]. In our recent studies, we showed that some of these compounds (berberine and coptisine) can act synergistically; thus, the multi-component composition of these extracts may significantly increase their pharmaceutical activity. Lans et al. showed that natural small-molecular compounds from *C. majus* (e.g., berberine, chelerythrine, sangunarine, and benzylisoquinoline alkaloids) are widely applied in traditional medicine to treat various gynecological disorders [28]. Some studies have demonstrated that a multi-component plant-derived product may be a potential remedy for endometriosis [9]. One of these medicines is a natural plant mixture GuiXiong Xiaoyi Wan (GXXYW), which contains a combination of 10 different plant extracts obtained from roots, tubers, whole plants, or seeds. Jin et al. found that treatment with the mixture significantly reduced the size of the endometrial explants in rats, which may have been caused by the inhibition of cell proliferation, an induction of apoptosis, or the regulation of the cell-mediated immune response [29]. Similarly, in our present study, we suggest that the source of a strong treatment effect of protoberberine-rich fraction (BBR) from *C. majus* is found in the complex composition/formulation of different natural, biologically active protoberberine compounds. Presumably, in endometriosis treatment, the efficacy is demonstrated via the anti-proliferative, antioxidant, and anti-inflammatory activities of the applied plant phytochemicals [30].

In the present research, we showed the regression of endometriosis loci after BBR supplementation in the diet of rats with experimentally induced endometriosis. We observed the removal of all visible ectopic endometriosis lesions in this group, which was confirmed by histological studies. In this work, in order to investigate the biological activity and effect of the studied protoberberine-rich formulation (BBR) from *C. majus* on endometriosis, NMR metabolomic analysis was performed. NMR analysis was used to investigate the metabolomic profiles of the blood serum of all of the studied groups of rats (E-BBR, E-C, and HC-SHO). Firstly, our analysis demonstrated metabolic changes between the E-BBR and E-C groups. We showed that the levels of energy metabolites (glucose and glutamine) were significantly altered, while the *p*-value for lactate of 0.08 was only slightly higher than the cutoff value (0.05). Furthermore, betaine was downregulated after BBR treatment. This comparison reveled that berberine and other protoberberine alkaloids may influence on endometriosis. In general, the serum glucose level reflects the balance between the entering and disposal processes happening in a living organism [31]. Glucose may undergo glycolysis, being converted in the glycolytic pathway to pyruvate, which may either be oxidized through the oxidative phosphorylation process (oxidative metabolism) or be transformed by lactate dehydrogenase to lactate. Additionally, glucose may be included in the pentose phosphate pathway or may be stored in the form of glycogen. In this study, glucose was at the highest level in the rats with no treated endometriosis (E-C), while the HC-SHO and E-BBR groups showed a similar concentration, showing that the protoberberine-rich fraction (BBR) caused a normalization effect on the glucose level. This could be because of two reasons—one being the limited appetite of the rats, and the second being accelerated glucose and/or pyruvate catabolism. This effect can be correlated with the dietary recommendation for endometriosis and endometrial cancer-suffering patients to limit sugar-rich food [32]. However, studies performed on women suffering from endometriosis have shown a reversal trend where glucose levels are lowered because of the accelerated/enhanced glucose metabolism, as well as from defects in the mitochondrial respiration. Furthermore, lactate was increased in endometriosis subjects/patients in comparison to healthy controls. Studies performed on endometrial tissue have revealed a glucose level dependence on disease progression, with the highest concentrations found at stage IV [25]. It is worth noting that endometriosis is associated with chronic inflammation. Overactivation of the immune response, altered immune signaling, increased levels of pro-inflammatory molecules, and increased blood levels of glucose and hyperinsulinemia can stimulate the growth of endometrial stromal cells [4].

The second metabolite that is crucial for cell bioenergetics is glutamine. Glutamine is a glucogenic amino acid necessary for the maintenance of ATP production and is essential for cell functional integrity. Several studies have demonstrated that glutamine favors cell growth, migration, and adhesion [33]. Therefore, glutamine can play an essential role in endometriosis development. Alterations in glutamine and glutamate levels are observed early in endometriosis patients [25]. In our studies, we found that the glutamine level was decreased in the E-BBR group in comparison to the E-C group. Based on these findings, we suggest that BBR can also modulate the serum level of glutamine. Zhang et al. indicated that BBR suppresses glutamine uptake and might have clinical potential as an anti-tumor drug [34].

In the present study, in the BBR treatment group, lactate was found to be at a higher level than in the control group, which was in contradiction to the above-presented results. Interestingly, the observed outcome was similar to the Warburg effect, when pyruvate conversion to lactate occurs during aerobic glycolysis. Together with the higher upregulation of lactate, a decreased glutamine level was detected. This effect could be associated with the metabolic outcome caused by berberine and its derivatives. Analyzing the TCA metabolites, the citrate level was at the same level in all of the investigated groups, while the malonate and pyruvate levels (despite not being statistically significant) were rather inversely correlated with glucose and glutamine. Therefore, it can be anticipated that the lactate level can be fueled by glutamine via malonic acid and pyruvate biosynthesis while simultaneously boosting the TCA cycle through 2-oxo-ketoglutarate. However, other reasons cannot be excluded. Moreover, in this study, we observed a decreased level of betaine after BBR treatment. Betaine (trimethylglycine) is known as an osmolyte and methyl-group donor, and BBR causes a lowering of its levels. Betaine plays important roles in the regulation of lipids and energy metabolism. Zhang et al. indicated that betaine inhibits fatty acid synthesis, but stimulates fatty acid oxidation and lipid secretion [35]. An alteration in the lipid profile was also demonstrated in our research. In comparison with the control group, the level of the L1 metabolite was decreased after treatment with protoberberine-rich fraction (BBR). This is in accordance with literature reports that berberine regulates serum lipid homeostasis and exerts a beneficial lipid-lowering effect. It has been shown that the oral administration of berberine significantly reduces cholesterol and triacylglycerols (TAGs). In addition, this natural product increases the activity of the AMP-activated protein kinase (AMPK) and promotes fatty acid β-oxidation.

The comparison between the metabolomic profiles of HC-SHO and E-BBR showed metabolic changes between the groups, which, in principle, should not exhibit the effect of endometriosis, where the metabolism of glucose was equilibrated; however, lactate was still at a lower level than in the reference control group. Similarly to previous comparisons, the betaine level was still lower than in the HC-SHO and EC groups. Additionally, the acetate, creatinine, and lipid L1 levels were perturbed. Acetate and creatine were downregulated, but their levels seemed to not be associated with BBR, because they were almost at the same level as that for the E-C group, while the L1 lipid level significantly declined after BBR treatment.

The last comparison of HC-SHO vs. E-C should reflect the changes caused by endometriosis. Out of all of the metabolites, there were only a few where glucose and lactate were perturbed together with isobutyrate, which can be generated by microbiota. According to previous scientific reports, berberine can modulate the gut microbiota by enriching short-chain fatty acid (SCFA)-producing bacteria and by reducing microbial diversity, which inhibits dietary polysaccharide degradation and decreases additional calorie intake in the gut, which may have beneficial effects on the host's metabolic status [36]. Gut microbiota play an important role in glucose and lipid homeostasis. Some studies have shown that the gut microbiome is altered in endometriosis. Ata et al. indicated that dysbiosis in the genital tract or gut microbiome can be associated with endometriosis [37]. Therefore, the modulation of the microbiota by berberine can improve the metabolic status of rats [38].

To sum up, the aim of this study was to investigate the effectiveness of a protoberberine-rich fraction in the treatment of endometriosis. One omics-based strategy, serum metabolomics profiling, was applied. The presented results indicate that the supplementation of rats with a protoberberine-rich fraction had a positive effect on the regulation of their metabolism—especially three metabolites, namely glucose, lactate, and glutamate—and inhibited the reoccurrence of endometriosis.

## 5. Conclusions

Berberine and other protoberberine alkaloids are characterized by strong pharmacological effects. Several studies showed that berberine modulates the glucose-lipid metabolism, the gut microbiota and inflammatory factors. Our metabolomics study confirmed its potential role in metabolic homoeostasis. In this study, we evaluated a natural protoberberine-rich formulation in the management of endometriosis and established a metabolomic profile after treatment. Our results suggest that the protoberberine-rich fraction obtained from the *C. majus* plant may be a promising agent for the treatment of endometriosis. We demonstrated the effect of the protoberberine-rich fraction on endometriotic lesion development, thus our findings can improve current preventive and therapeutic strategies for endometriosis.

## Figures and Tables

**Table 1:** The MVA models summary for EC and HC-SHO comparison.

Comparision	Model Type	PC/LV	N=	R^2^X(cum)	R^2^Y(cum)	Q^2^(cum)	CV-ANOVA *p* Value
EC vs. HC-SHO	OPLS-DA	2	12	0.584	0.85	0.771	2.127 × 10^−2^

**Table 2:** Summary of univariate analysis for EC and HC-SHO comparison.

Metabolite	Mean Relative Integral Value EC	Mean Relative Concentration HC-SHO	RSD EC (%)	RSD HC-SHO (%)	*p*-Value	PD %
Glucose ^(a)^	3.81	2.54	10.77	21.09	4.04 × 10^−3^	39.86%
Lactate ^(a)^	0.89	1.64	14.07	39.36	1.616 × 10^−2^	−58.97%
Isobutyrate ^(b)^	0.02	0.03	19.45	13.44	4.48 × 10^−2^	−20.70%

^(a)^ U test; ^(b)^ *t*-test for equal variances; PD%: percentage difference.

**Table 3:** Summary of univariate analysis for EC and E-BBR comparison.

Metabolite	Mean Relative Integral Value EC	Mean Relative Concentration E-BBR	RSD EC (%)	RSD E-BBR (%)	*p*-Value	PD %
Glucose ^(a)^	2.29	3.81	43.02	10.77	2.197 × 10^−3^	49.69%
Glutamine ^(a)^	0.15	0.20	26.17	2.94	1.098 × 10^−3^	27.89%
Betaine ^(b)^	0.50	0.66	24.63	11.52	3.192 × 10^−2^	27.23%

^(a)^ U test; ^(b)^ *t*-test for equal variances; PD%: percentage difference.

**Table 4:** Summary statistical analysis for HC-SHO vs. E-BBR comparison.

Metabolite	Mean Relative Integral Value HC-SHO	Mean Relative Integral Value E-BBR	RSD HC-SHO (%)	RSD E-BBR (%)	*p*-value	PD %
Acetate ^(b)^	0.13	0.18	33.51	28.06	2.784 × 10^−2^	32.76%
Betaine ^(b)^	0.50	0.64	24.63	27.82	4.979 × 10^−2^	24.74%
Creatine ^(a)^	0.14	0.29	34.59	96.40	2.515 × 10^−2^	67.76%
L1 ^(b)^	6.62	8.95	36.37	20.86	3.345 × 10^−2^	29.88%

^(a)^ U test; ^(b)^ *t*-test for equal variances; PD%: percentage difference.

## Data Availability

All support data used in this study are available from the authors.

## Supplementary Materials

The following are available online at https://www.mdpi.com/article/10.3390/pharmaceutics13070931/s1, Figure S1: HPLC/MS Extracted Ion Chromatogram of BBR. Figure S1a: Full HPLC chromatogram of protoberberine-rich fraction from *C. majus*; UltiMate 3000 UHPLC System. Figure S2: ESI-MS spectra of alkaloids from BBR after HPLC separation (the retention time range from 4 to 10 min). Figure S3: ESI-MS spectra of alkaloids from BBR after HPLC separation (the retention time range from 9.4 to 13 min). Table S1: HPLC/MS data for the alkaloids, isolated from the BBR of *C. majus* ethanolic extract. Figure S4: Accurate MS2 spectrum of the parent ion *m*/*z* 370, molecular [M+H]^+^ ion of the *C. majus* alkaloid with RT 9.5 min. Collision energy 35 eV. Figure S5: Accurate MS2 spectrum of the parent ion *m*/*z* 354, molecular [M+H]^+^ ion of the *C. majus* alkaloid with RT 9.2 min. Collision energy 35 eV. Figure S6: Accurate MS2 spectrum of the parent ion *m*/*z* 354, molecular [M+H]^+^ ion of the *C. majus* alkaloid with RT 8.3 min. Collision energy 35 eV. Figure S6a: Accurate MS2 spectrum of the parent ion *m*/*z* 338, molecular [M+H]^+^ ion of the *C. majus* alkaloid with RT 9.8 min. Collision energy 35 eV. Figure S7: The representative ^1^H NMR spectrum (DMSO-*d*_6_, 800 MHz) of the protoberberine-rich fraction from *Chelidonium majus* L. extract. Table S2: ^1^H and ^13^C NMR chemical shifts of metabolites identified in protoberberine-rich fraction from *Chelidonium majus* L. extract. Table S3: Metabolite assignments of major resonances detected in ^1^H NMR spectra from serum samples of rates.
