# Supplementary material for: Effect of Protoberberine-Rich Fraction of Chelidonium majus L. on Endometriosis Regression
Source: Pharmaceutics. 2021 Jun 23;13(7):931. doi: 10.3390/pharmaceutics13070931 (PMC8309065; doi:10.3390/pharmaceutics13070931)
Supplement: Supplementary file 1 [file pharmaceutics-13-00931-s001.zip › pharmaceutics-1240991-SI.pdf]

pharmaceutics

Supplementary Materials: Effect of Protoberberine-Rich Fraction of *Chelidonium majus* L. on Endometriosis Regression

Alicja Warowicka, Badr Qasem, Anna Dera-Szymanowska, Maria Wołuń-Cholewa, Patryk Florczak, Nikodem Horst, Marta Napierała, Krzysztof Szymanowski, Łukasz Popenda, Grażyna Bartkowiak, Ewa Florek, Anna Goździcka-Józefiak, and Piotr Młynarz

1. Identification of Compounds by HPLC-ESI/MS and NMR

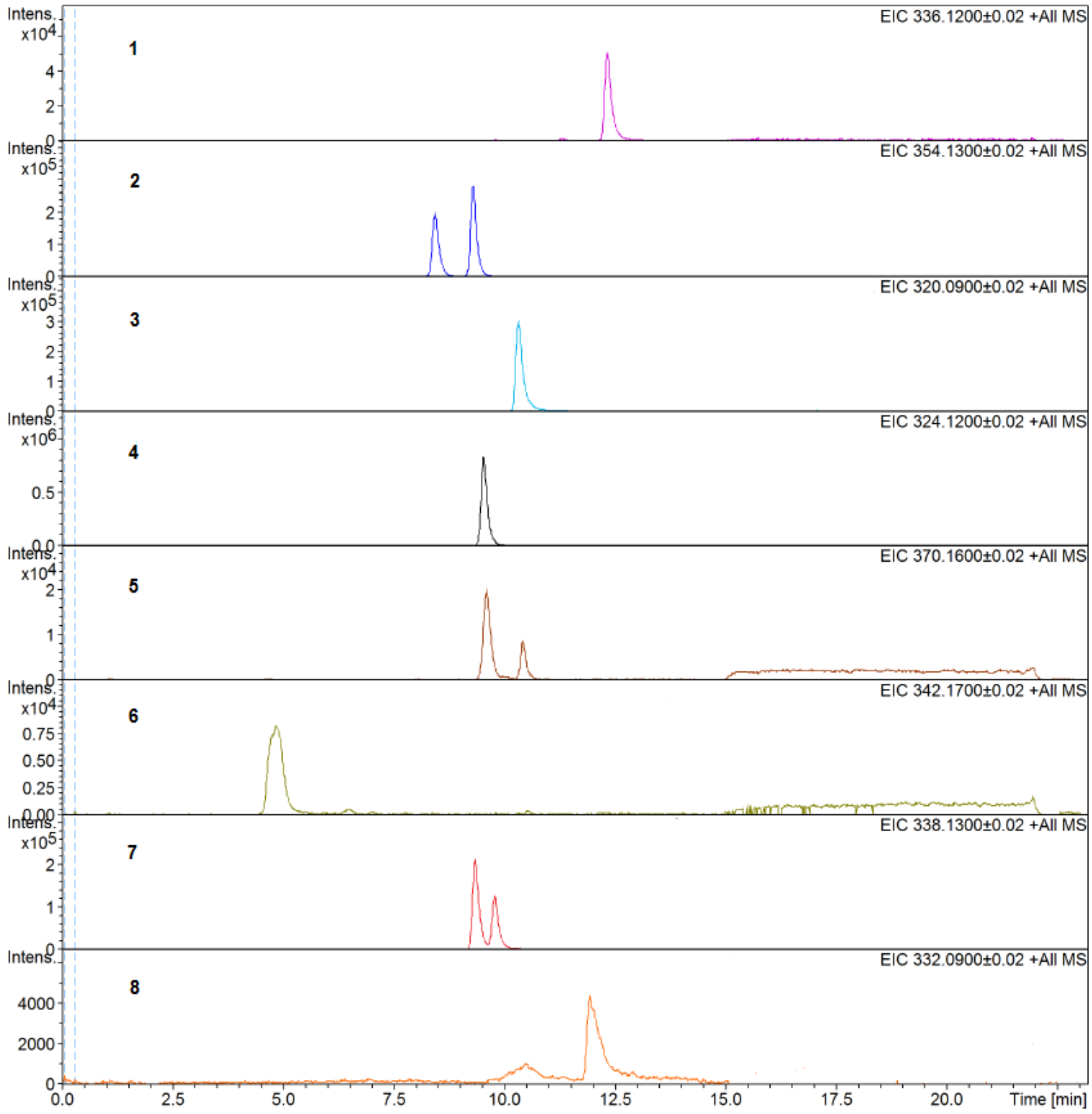

Figure S1. 1 - berberine, 2 – protopine + chelidonine, 3 - coptisine, 4 - stylopine, 5 - allocryptopine + unidentified isomeric alkaloid, 6 - magnoflorine, 7 - dihydroberberine + unidentified isomeric alkaloid, 8 - sanguinarine.

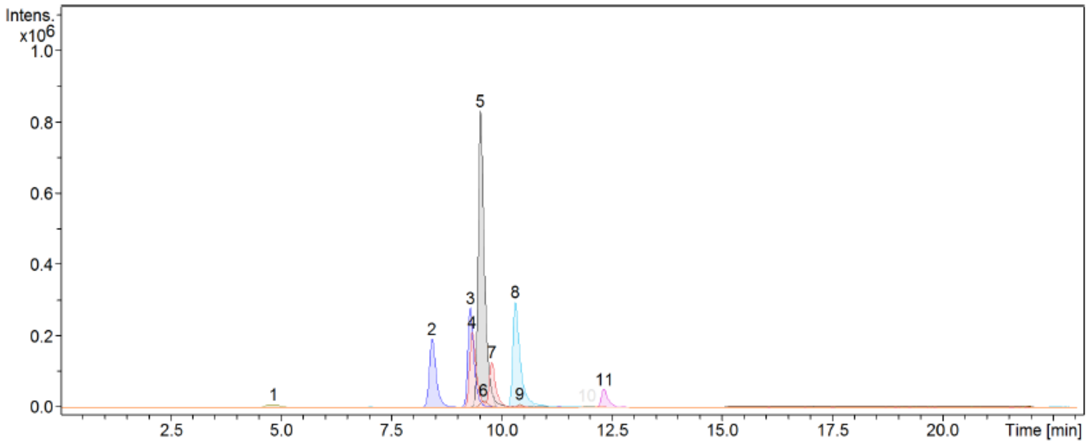

Figure S1a. Full HPLC chromatogram of protoberberine-rich fraction from *C. majus*; UltiMate 3000 UHPLC System.

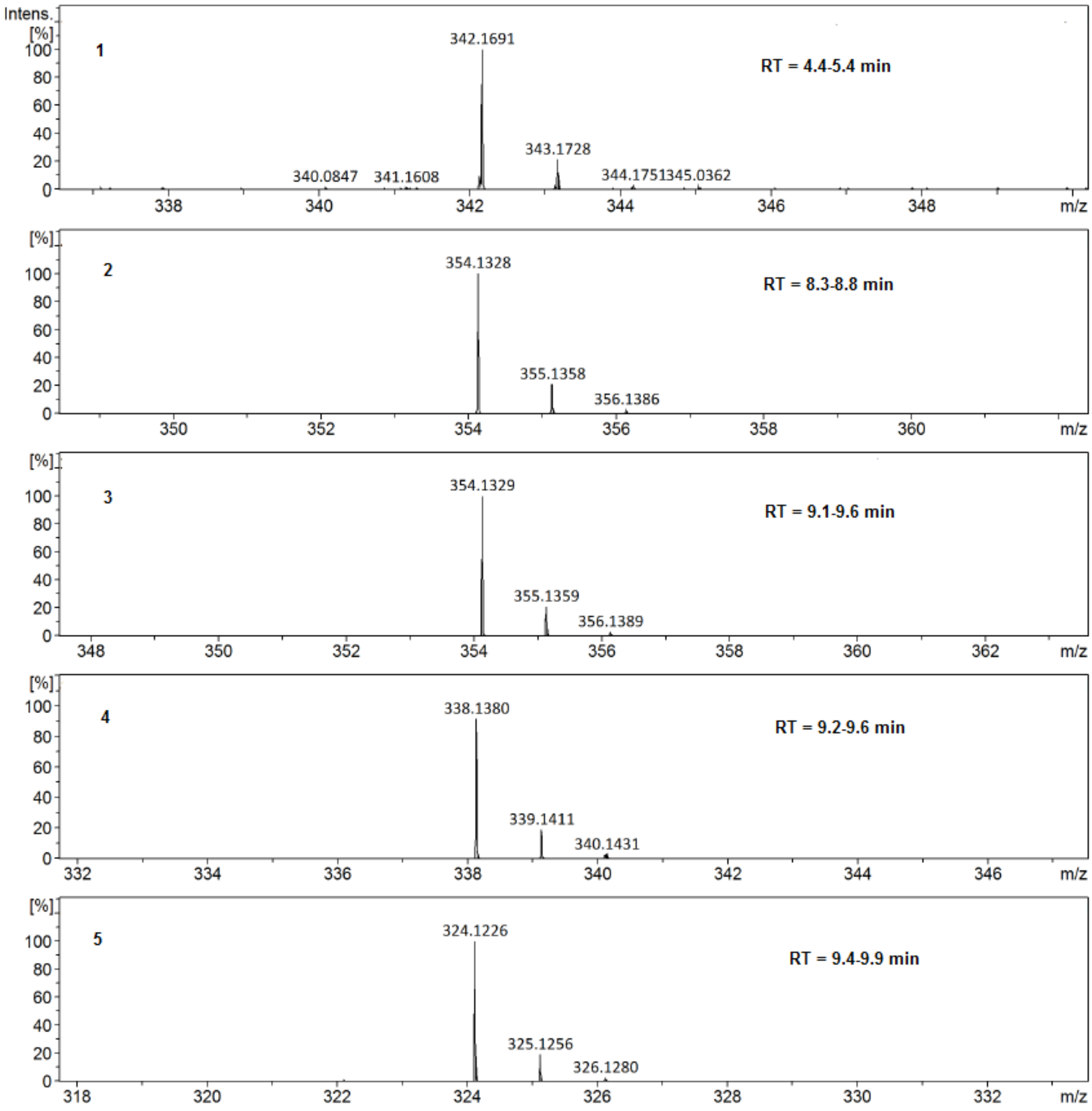

Figure S2. ESI-MS spectra of alkaloids from BBR after HPLC separation, obtained in the retention time range from 4 to 10 min – zoom showing molecular ions with isotopic distribution; HPLC retention time (RT) of each alkaloid is given in the drawing.

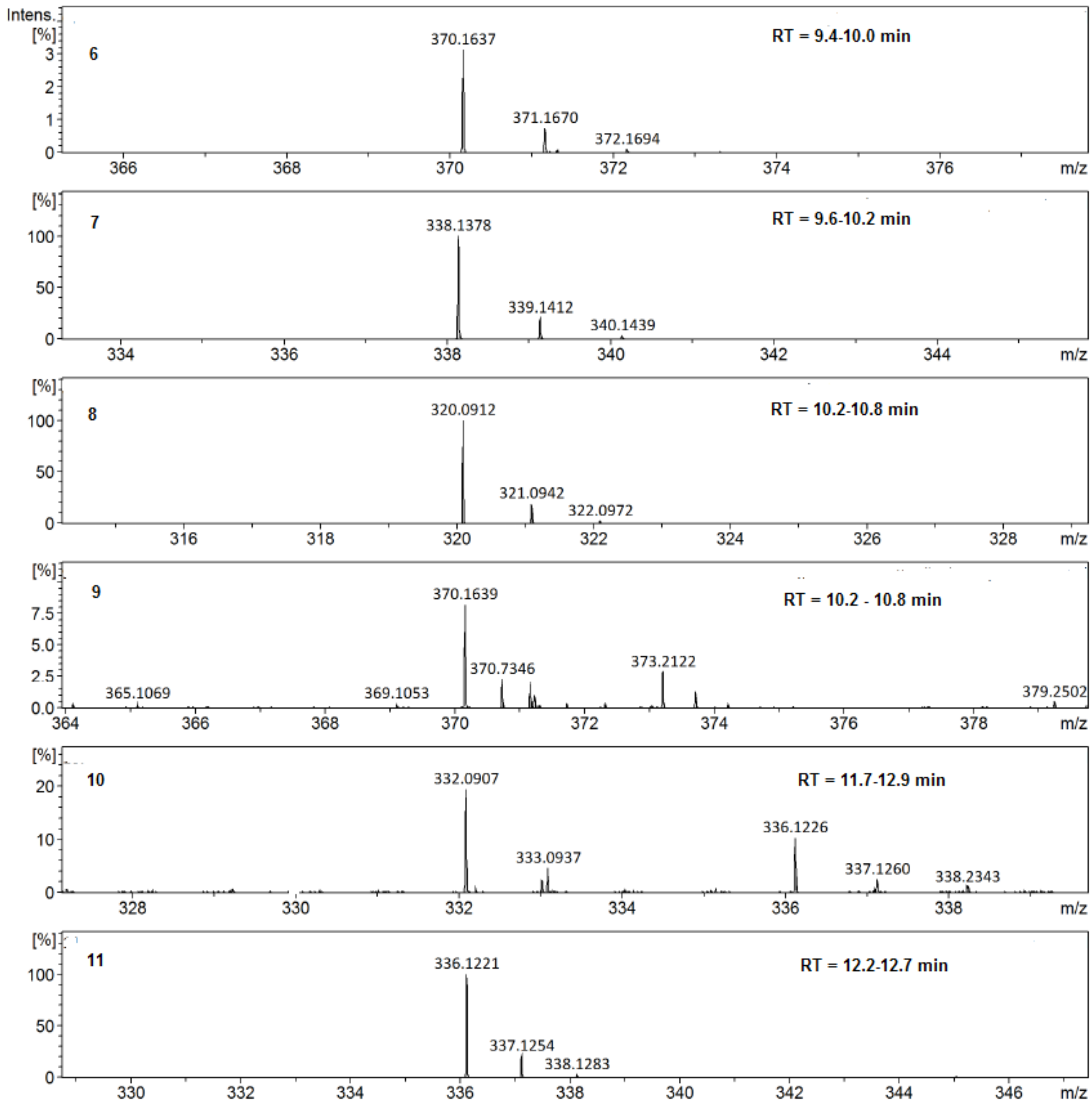

Figure S3. ESI-MS spectra of alkaloids from BBR after HPLC separation, obtained in the retention time range from 9.4 to 13 min – zoom showing molecular ions with isotopic distribution; HPLC retention time (RT) of each alkaloid is given in the drawing.

Table S1. HPLC/MS data for the alkaloids, isolated from the BBR of *C. majus* ethanolic extract – retention times in HPLC, *m/z* values for molecular ions observed and calculated from the molecular formulae assigned to the respective alkaloids, and alkaloids names.

Comp. no	Retention Time RT [min]	*m/z* Observed	Formula of the Ion, Observed in MS Spectrum	*m/z* Calculated	Alkaloid
1	4.8	342.1691	$C_{20}H_{24}NO_4^+$	342.1706	magnoflorine
2	8.3	354.1328	$[C_{20}H_{19}NO_5+H]^+$	354.1342	protopine
3	9.2	354.1329	$[C_{20}H_{19}NO_5+H]^+$	354.1342	chelidonine
4	9.3	338.1480	$[C_{20}H_{19}NO_4+H]^+$	338.1393	isomer of comp.7
5	9.4	324.1226	$[C_{19}H_{17}NO_4+H]^+$	324.1236	stylopine
6	9.5	370.1637	$[C_{21}H_{23}NO_5+H]^+$	370.1655	allocryptopine
7	9.8	338.1378	$[C_{20}H_{19}NO_4+H]^+$	338.1393	dihydroberberine
8	10.3	320.0912	$C_{19}H_{14}NO_4^+$	320.0923	coptisine
9	10.6	370.1639	$C_{21}H_{24}NO_5^+$	320.0923	isomer of comp. 6
10	11.9	332.0907	$C_{20}H_{14}NO_4^+$	332.0923	sanguinarine
11	12.5	336.1236	$C_{20}H_{18}NO_4^+$	336.1236	berberine

Compounds listed in the Table S1: 2 (protopine), 3 (chelidonine) and 6 (allocryptopine) have been identified based on the accurate *m/z* values of their $[M+H]^+$ ions and MS2 fragmentation patterns. The identity of 5 (stylopine), 8 (coptisine), 10 (sanguinarine) and 11 (berberine) were additionally confirmed through the ^{1}H NMR spectra. Assignment of compound 1 as magnoflorine was based on the paper by Jeong and Lim [3], who performed the UPLC-MS experiment in similar conditions as used in the present article (C18 column, 0.1% formic acid with acetonitrile as eluents) and separated magnoflorine as a component with the shortest retention time of all *C. majus* alkaloids and molecular ion $[M]^+$ *m/z* 342.1704, which is in accordance with the formula of a cation $[C_{20}H_{24}NO_4]^+$. Moreover, several paper report the presence of magnoflorine in *Chelidonium majus* L. extracts [4–7] as a minor alkaloid.

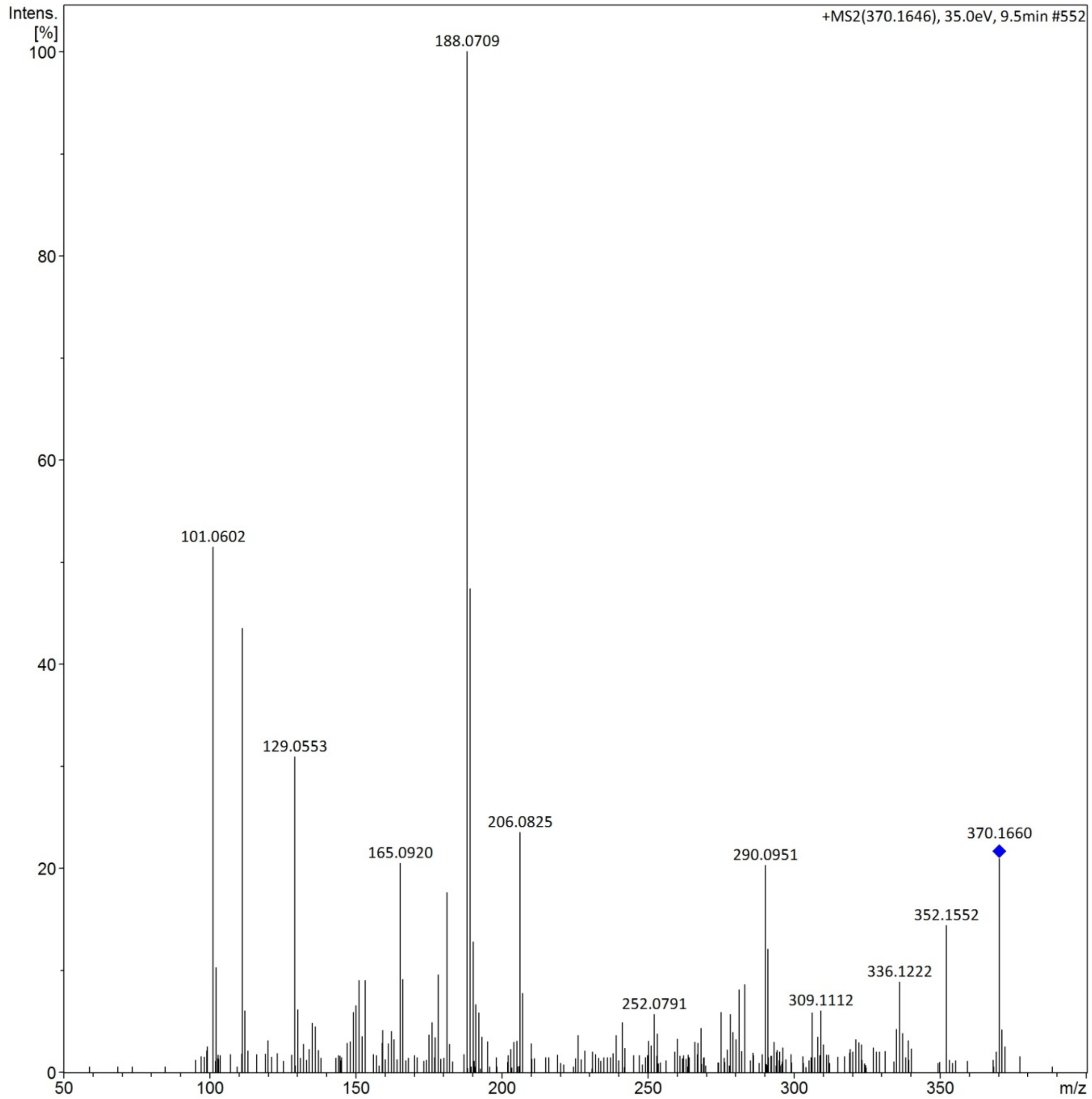

Figure S4. Accurate MS2 spectrum of the parent ion *m/z* 370, molecular $[M+H]^+$ ion of the *C. majus* alkaloid with RT 9.5 min. Collision energy 35 eV.

Identification of allocryptopine is based on the comparison of MS2 spectrum of the $[M+H]^+$ ion *m/z* 370, corresponding to the molecular ion of compound with retention time 9.5 min with the literature data [1]. The authors report that the allocryptopine parent ion *m/z* 370 produces under the MS2 experiment following product ions: 370.1664→352.1554, 290.0952, 206.0821, 189.0782, 188.0712, 165.0914. The *m/z* values obtained in our research are respectively (see the MS2 spectrum in Figure S4): 370.1660→352.1552, 290.0951, 206.0825, 188.0709, 165.0920, which confirms that the compound with retention time 9.5 min is allocryptopine.

Discrimination between chelidonine and protopine was possible also on the basis of their MS2 spectra. There are two compounds with molecular ions at the same *m/z* 354, one appearing at retention time 8.3 min and the second at RT = 9.2 min. According to the chromatogram 2 in Figure S1, the content of compound with RT 9.2 min in the extract is bigger than the one with RT 8.3 min. The MS2 spectra of the $[M+H]^+$ ion, *m/z* 354, for these two compounds differ significantly (Figure S5, S6).

MS2 data for chelidonine are placed in MassBank, Record NA 002674 [2], where the most important product ions for the parent ion at *m/z* 354 are: 354.1335→323.0913, 305.0807, 275.0702, 247.0735, 163.0388, 135.044. The respective values are seen in Figure

S5 for the compound of RT = 9.2 min, i.e. 354.1341→323.0925, 305.08, 275.0708, 247.0757, 163.0395, 135.0445. The above data indicate that alkaloid of RT 9.2 min is chelidonine.

According to the literature [1], the MS2 product ions of protopine molecular ion *m*/*z* 354 are: 354.1342→336.1226, 275.0702, 206.0808, 189.0777, 149.0593. These values are in agreement with the product ions shown in Figure S6 (RT 8.3 min): 354.1335→336.1243, 275.0712, 206.0819, 189.0787, 149.0602, so the compound at RT 8.3 min can be with the great probability identified as protopine.

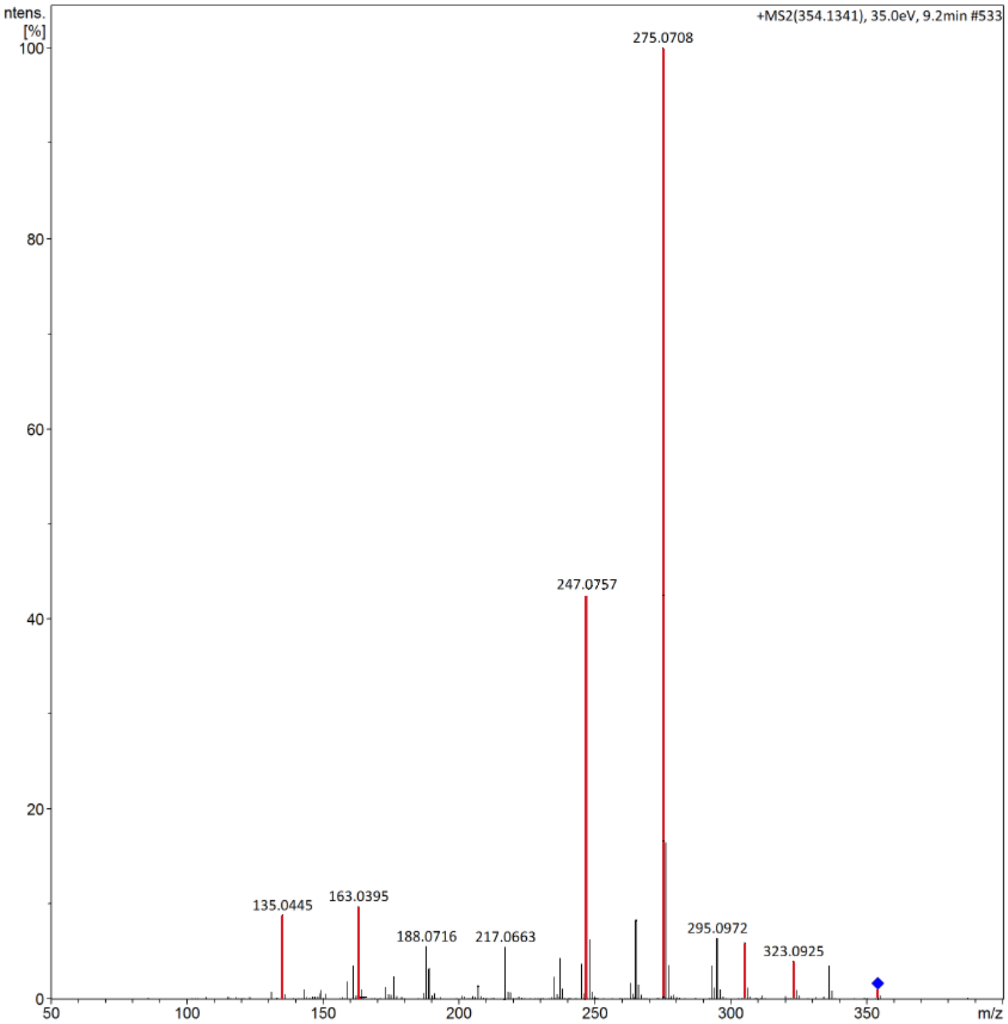

Figure S5. Accurate MS2 spectrum of the parent ion *m*/*z* 354, molecular $[M+H]^+$ ion of the *C. majus* alkaloid with RT 9.2 min. Collision energy 35 eV.

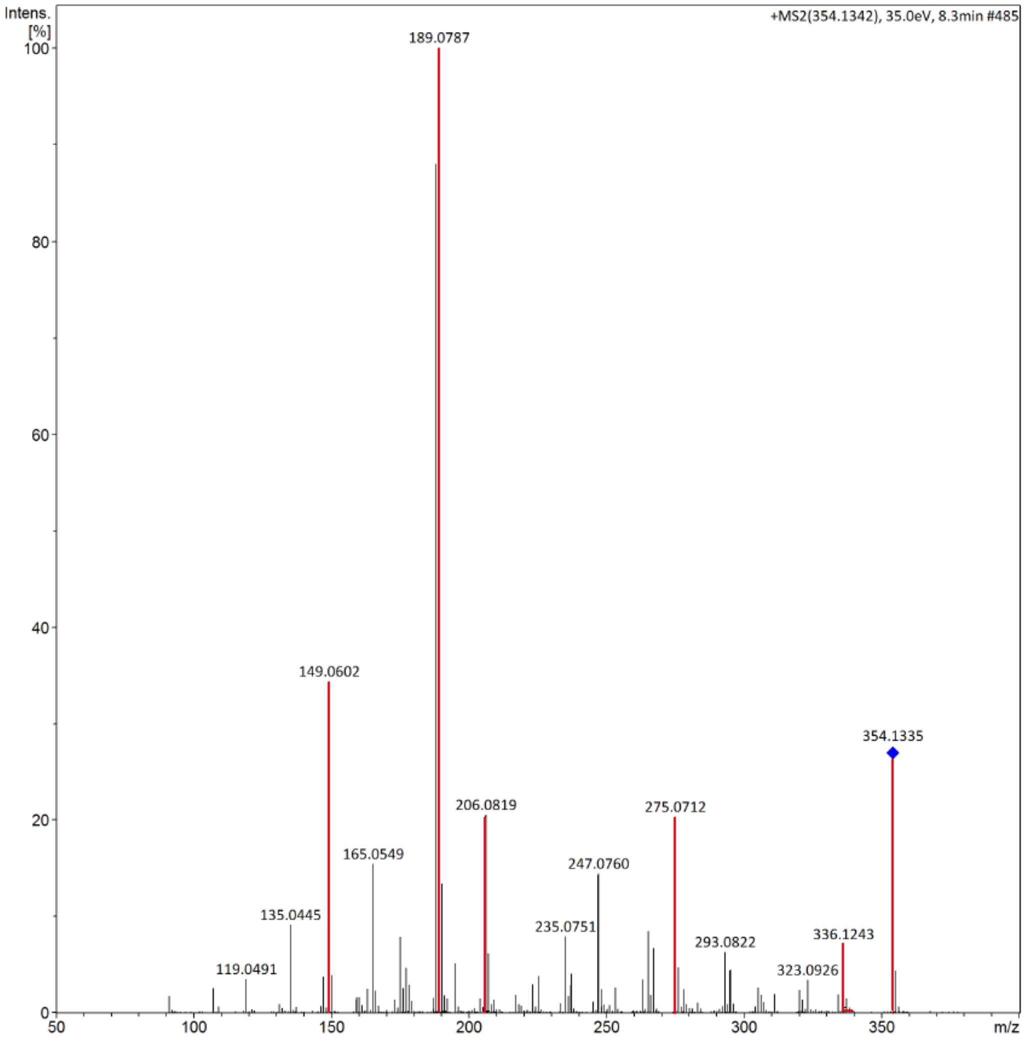

Figure S6. Accurate MS2 spectrum of the parent ion *m/z* 354, molecular $[M+H]^+$ ion of the *C. majus* alkaloid with RT 8.3 min. Collision energy 35 eV.

Compound 7 (RT = 9.8 min, *m/z* 338.1378] has been identified as dihydroberberine accordingly to the identification described in [3]. Its retention time (9.8 min) is very close to the dihydroberberine RT (9.57 min) reported by Jeong and Lim [3] and the MS2 spectrum of the ion at *m/z* 338.138 is identical in [3] with obtained by us (Figure S6a).

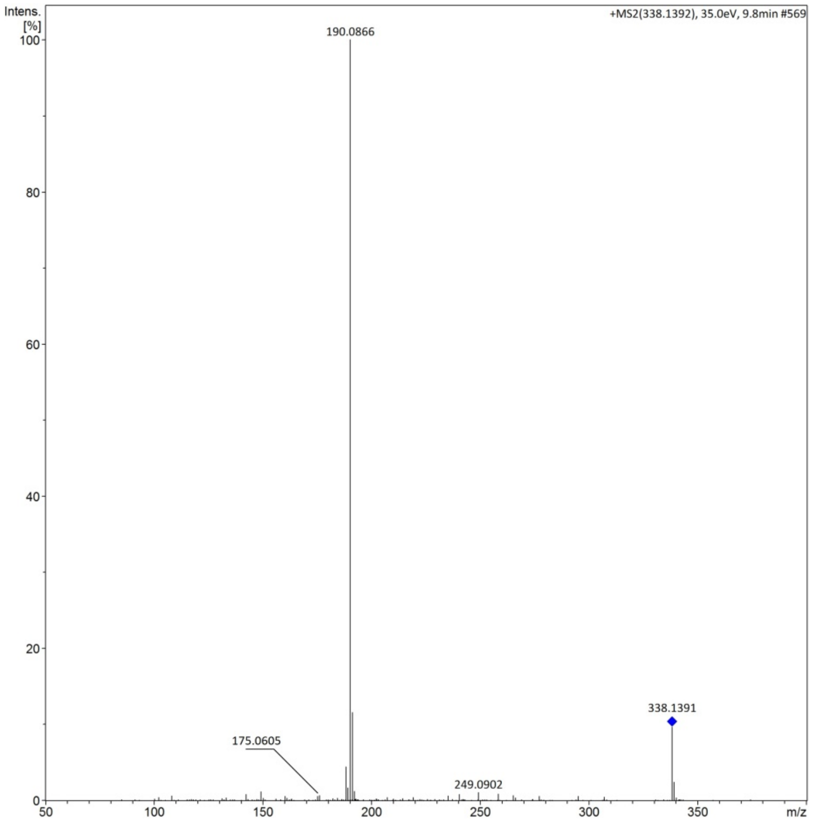

Figure S6a. Accurate MS2 spectrum of the parent ion *m/z* 338, molecular $[M+H]^+$ ion of the *C. majus* alkaloid with RT 9.8 min. Collision energy 35 eV.

The same value of molecular ion *m/z* 338.138 shows compound 4, i.e. the elemental composition of the ion 4 is the same as 7 ($C_{20}H_{20}NO_4^+$). The compositions $C_{20}H_{20}NO_4^+$ or $[C_{20}H_{19}NO_4+H]^+$ correspond to the molecular formula of several protoberberine alkaloids except dihydroberberine, for example columbamine, jatrorrhizine or N-methylstylopine, however, based on the obtained data, it was not possible to establish the structure of the compound 4 with certainty.

Compound 9, RT = 10.6 min, *m/z* 370.1639, corresponds to the elemental composition $C_{21}H_{24}NO_5^+$, so it can be an $[M+H]^+$ ion $[C_{21}H_{23}NO_5+H]^+$ of allocryptopine isomer. There are numerous reports that allocryptopine is often found alongside its minor isomeric alkaloid cryptopine, for example in Papaver coreanum [8] and other plants of Papaveraceae family. The content of compound 9 in protoberberine-rich fraction of *C. majus* is much lower than that of 6 (allocryptopine), but its structure has not been proven. Nevertheless, it may be suggested that compounds 6 and 9 are isomeric.

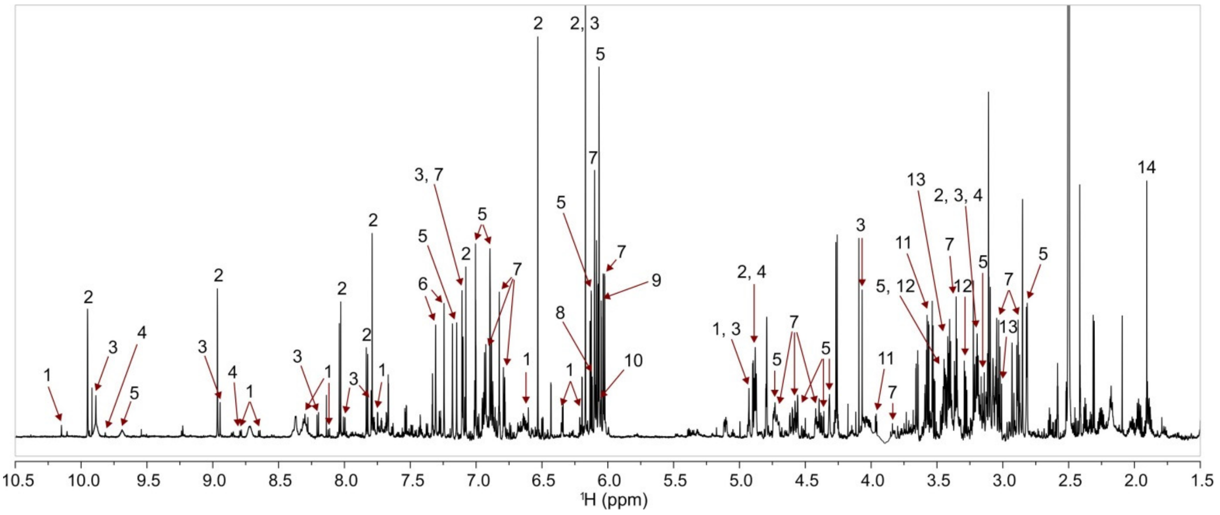

Figure S7. The representative ^{1}H NMR spectrum (DMSO-d_6, 800 MHz) of the protoberberine-rich fraction from *Chelidonium majus* L. extract. The following compounds are identified: 1, Sanguinarine; 2, Coptisine; 3, Berberine; 4, Unidentified protoberberine alkaloid; 5, Chelidonine; 6, NH_4^+; 7, Stylopine; 8, Unidentified alkaloid; 9, Unidentified alkaloid; 10, Unidentified alkaloid; 11, Glyceric acid; 12, Glycerol; 13, Ethanol; 14, Acetate.

Table S2. ^{1}H and ^{13}C NMR chemical shifts of metabolites identified in protoberberine-rich fraction from *Chelidonium majus* L. extract.

**Metabolites.**	**δ¹H**	**δ¹³C ***
Acetate	1.91	172.0, 20.7
Berberine	9.89, 8.95, 8.20, 8.00, 7.80, 7.09, 6.17, 4.93, 4.09, 4.07, 3.20	150.4, 149.9, 147.7, 145.2, 143.6, 137.6, 132.9, 130.7, 123.5, 121.2, 120.5, 120.0, 108.4, 105.6, 61.8, 56.8, 55.1, 26.2
Chelidonine	9.69, 7.15, 7.01, 6.90, 6.13, 6.07, 6.07, 4.73, 4.60, 4.37, 4.32, 3.44, 3.15, 3.06, 2.82	149.1, 145.7, 145.3, 142.9, 129.7, 128.6, 120.9, 119.4, 111.8, 108.7, 108.4, 101.7, 101.4, 70.1, 61.8, 51.0, 40.2, 38.7, 36.9
Coptisine	9.95, 8.97, 8.03, 7.83, 7.79, 7.08, 6.53, 6.17, 4.88, 3.20	149.8, 147.8, 147.0, 144.4, 143.8, 136.8, 132.4, 130.6, 121.5, 121.0, 120.9, 120.4, 111.6, 108.3, 105.2, 104.4, 101.9, 55.0, 26.1
Glyceric acid	3.96, 3.55	174.0, 71.7, 63.5
Glycerol	3.45, 3.35, 3.28	72.3, 62.9
NH_4^+	7.24	
Sanguinarine	10.15, 8.79, 8.65, 8.31, 8.28, 8.12, 7.76, 6.61, 6.35, 4.93	150.0, 148.6, 132.2, 131.2, 109.5, 52.0
Stylopine	7.11, 6.93, 6.82, 6.79, 6.10, 6.03, 6.02, 4.73, 4.57, 4.41, 3.79, 3.36, 3.07, 3.02, 2.88	121.1, 107.9, 105.2, 101.0, 58.9, 49.4, 32.0, 24.9

* Due to the low concentration of certain components data for which the identification was reliable were presented.

2. Metabolomic Analysis. Identification of Metabolites

Table S3. Metabolite assignments of major resonances detected in ^{1}H NMR spectra from serum samples of rates. Chemical shifts (δ) used for identification of compounds are reported and peak multiplicity. Chemical shifts were referenced to the H1 of Glucose (δ = 5.222).

Nr	Metabolites	Chemical Shift [ppm]	Multiplicity	HMDB ID
1	Formate	8.441	s	HMDB0000142
2	Histidine	7.736	s	HMDB0000177
3	Phenylalanine	7.412	m	HMDB0000159
4	Tyrosine	6.879	m	HMDB0000158
5	Cytidine	6.048	d	HMDB0000089
6	Urea	5.759	s	HMDB0000294
7	L-1 _ VLDL	0.84		-
8	Glucose	5.222	d	HMDB0000122
9	Unknown_1	4.4	s	-
10	Lactate	4.101	dd	HMDB0000190
11	Glycerol	3.551	m	HMDB0000131
12	Proline	3.325	m	HMDB0000162
13	Betaine	3.251	s	HMDB0000043
14	Choline	3.188	s	HMDB0000097
15	Malonate	3.139	s	HMDB0000691
16	Creatinine	3.029	s	HMDB0000562
17	Creatine	3.024	s	HMDB0000064
18	Lysine	3.014	t	HMDB0000182
19	Dimethylamine	2.715	s	HMDB0000087
20	Citrate	2.671	d	HMDB0000094
21	Methionine	2.628	m	HMDB0000696
22	Glutamine	2.419	m	HMDB0000641
23	Pyruvate	2.358	s	HMDB0000243
24	Acetone + lipid	2.222	s	HMDB0001659
25	Acetate	1.904	s	HMDB0000042
26	L-2_ VLDL	1.55		-
27	Alanine	1.463	d	HMDB0000161
28	L-3_ LDL	1.223		-
29	3-Hydroxybutyrate	1.184	d	HMDB0000011
30	Isobutyrate	1.056	d	HMDB0001873
31	Valine	1.026	d	HMDB0000883
32	Isoleucine	0.994	d	HMDB0000172
33	Leucine	0.94	m	HMDB0000687
34	L-4_LDL	0.84		-

s (singlet), d (doublet), t (triplet), q (quartet), dd (doublet doublet), m (multiple signals), VLDL (very low-density lipoprotein) and LDL (low-density lipoprotein).

References

[1] Huang, Y.J.; Xiao, S.; Sun, Z.L.; Zeng, J.G.; Liu, Y.S.; Liu, Z.Y. Identification of allocryptopine and protopine metabolites in rat liver S9 by high-performance liquid chromatography/quadrupole-time-of-flight mass spectrometry. Rapid Commun Mass Spectrom. 2016, 30(13), 1549-59.
[2] https://massbank.eu/MassBank/RecordDisplay.jsp?id=NA002674
[3] Jeong W. T.; Lim H. B.. Determination of isoquinoline alkaloids by UPLC-ESI-Q-TOF MS: Application to Chelidonium majus L. Analytical Science & Technology, 2017, 30 (6), 379-389.
[4] Slavik J, Slavikova L. Minor alkaloids from Chelidonium majus L. Collect. Czechoslov. Chem. Commun. 1977; 42:2686-93.
[5] Kędzia B., Łożykowska K., Gryszczyńska A.; Skład chemiczny i zawartość substancji biologicznie aktywnych w Chelidonium majus. Postępy fitoterapii 2013, 3, 174-181. (Polish)
[6] Wu C.; Wang X.; Xu M.; Liu Y.; Di X. Intracellular accumulation as an indicator of cytotoxicity to screen hepatotoxic components of Chelidonium majus L. by LC-MS/MS. Molecules 2019, 24, 2410.
[7] Hădărugă D. I.; Hădărugă N. G. Antioxidant activity of Chelidonium majus L. extracts from the Banat county. J. Agroalimentary Proc. Technol. 2009, 15 (3), 396-402.
[8] Lee D.-U.; Park Y. H.; Wessjohann L.; Schmidt J. Alkaloids from Papaver coreanum. Natural Products Communications 2011, 6 (11), 1593-1594.
